# A deep learning model for prediction of post hepatectomy liver failure after hemihepatectomy using preoperative contrast-enhanced computed tomography: a retrospective study

**DOI:** 10.3389/fmed.2023.1154314

**Published:** 2023-06-28

**Authors:** Xiaoqing Xu, Zijian Xing, Zhiyao Xu, Yifan Tong, Shuxin Wang, Xiaoqing Liu, Yiyue Ren, Xiao Liang, Yizhou Yu, Hanning Ying

**Affiliations:** ^1^Department of Nursing, Sir Run Run Shaw Hospital, Zhejiang University School of Medicine, Hangzhou, China; ^2^Deepwise Artificial Intelligence Laboratory, Hangzhou, China; ^3^Department of Pathology, Sir Run Run Shaw Hospital, Zhejiang University School of Medicine, Hangzhou, China; ^4^Department of General Surgery, Sir Run Run Shaw Hospital, Zhejiang University School of Medicine, Hangzhou, China; ^5^Faculty of Engineering, The University of Hong Kong, Hong Kong, China

**Keywords:** deep learning, hemihepatectomy, liver failure, prediction model, contrast – enhanced CT

## Abstract

**Objective:**

Post-hepatectomy liver failure (PHLF) remains clinical challenges after major hepatectomy. The aim of this study was to establish and validate a deep learning model to predict PHLF after hemihepatectomy using preoperative contrast-enhancedcomputed tomography with three phases (Non-contrast, arterial phase and venous phase).

**Methods:**

265 patients undergoing hemihepatectomy in Sir Run Run Shaw Hospital were enrolled in this study. The primary endpoint was PHLF, according to the International Study Group of Liver Surgery’s definition. In this study, to evaluate the proposed method, 5-fold cross-validation technique was used. The dataset was split into 5 folds of equal size, and each fold was used as a test set once, while the other folds were temporarily combined to form a training set. Performance metrics on the test set were then calculated and stored. At the end of the 5-fold cross-validation run, the accuracy, precision, sensitivity and specificity for predicting PHLF with the deep learning model and the area under receiver operating characteristic curve (AUC) were calculated.

**Results:**

Of the 265 patients, 170 patients with left liver resection and 95 patients with right liver resection. The diagnosis had 6 types: hepatocellular carcinoma, intrahepatic cholangiocarcinoma, liver metastases, benign tumor, hepatolithiasis, and other liver diseases. Laparoscopic liver resection was performed in 187 patients. The accuracy of prediction was 84.15%. The AUC was 0.7927. In 170 left hemihepatectomy cases, the accuracy was 89.41% (152/170), and the AUC was 82.72%. The accuracy was 77.47% (141/182) with liver mass, 78.33% (47/60) with liver cirrhosis and 80.46% (70/87) with viral hepatitis.

**Conclusion:**

The deep learning model showed excellent performance in prediction of PHLF and could be useful for identifying high-risk patients to modify the treatment planning.

## 1. Introduction

Liver resections are performed for both benign and malignant liver diseases. Hemihepatectomy is one type of major liver resection for the treatment of liver disease (1–3). Posthepatectomy liver failure (PHLF) is the most worrisome complication after major hepatectomy and is the leading cause of postoperative mortality (4–8). Previous reports have shown that the incidence of PHLF after liver resection varies and ranges from 0.7 to 39.6% (9, 10). Moreover, PHLF is the major cause of prolonged hospitalization, increased costs, and poor long-term outcomes in patients undergoing hepatectomy. In 2011, the International Study Group of Liver Surgery (ISGLS) proposed a standardized definition and severity grading for PHLF. The grade A, B, and C definitions are associated with mortality rates of 0, 12 and 54%, respectively, (7, 11).

The prediction of PHLF before hemihepatectomy should be a major concern for hepatobiliary surgeons and patients. A tool to accurately predict the risk for PHLF preoperatively will assist with patient selection and earlier intervention to potential PHLF patients. Currently, there are several predictors of PHLF reported, such as indocyanine green (ICG) clearance (12, 13), “50–50 Criteria” (4, 14), model for end-stage liver disease (MELD) system (15), Child-Pugh grade (16) and Future liver remnant (FLR) volume (17, 18). In addition, multivariable models are created to predict the risk of PHLF. But, there is still no standard model for clinical application due to limitations of each current models.

Deep learning with convolutional neural networks (CNNs) has been proven to have clinical significance in various medical image interpretation tasks, such as identifying and grading diabetic retinopathy, classifying skin lesions, classifying liver masses as benign or malignant and grading breast nodules based on BI-RADS, with accuracy comparable to experts (19–23). Recently, a research showed that deep learning model, based on medical data, could be used for preoperative prediction of severe liver failure after hemihepatectomy in patients with hepatocellular carcinoma (24). But there was no study on deep learning for preoperative prediction of PHLF with liver images.

Contrast-enhanced computed tomography (CT) is a common examination for the assessment of liver disease, because the vascularity and contrast agent enhancement patterns of liver lesions provide useful information for evaluation. A previous study reported that a nomogram combining CT image, serum albumin (Alb) and serum total bilirubin (Tbil) showed a good performance for PHLF preoperative prediction in patients with resectable HCC (25).

In this study, we aimed to investigate the prediction performance of deep learning model for PHLF after hemihepatectomy on preoperative contrast-enhanced CT images.

## 2. Methods

### 2.1. Patients

This retrospective study was approved by the Institutional Review Board of Sir Run Run Shaw Hospital (SRRSH). Informed consent was waived.

Between January 2017 and December 2021, consecutive patients who underwent hemihepatectomy at SRRSH were reviewed retrospectively. A total of 266 patients who met the inclusion criteria were enrolled. The inclusion criteria were as follows: (1) patients who underwent hemihepatectomy, (2) Patients above the age of 14, and (3) patients who underwent contrast-enhanced CT and serum liver function and coagulating function testing within 1 week before operation. The exclusion criteria were as follows: (1) patients with any antitumor therapy before surgery and (2) patients who had minor liver resections or more than hemihepatectomy.

### 2.2. Data collection and imaging quality control

Preoperative data were collected, including age, gender, the presence of viral hepatitis and liver cirrhosis, grade of Child-Pugh, pathological diagnosis, American Society of Anesthesiologists Score (ASA score) and preoperative contrast-enhanced CT image. Perioperative laboratory data were recorded, including total bilirubin (Tbil), international normalized ratio (INR) before operation and on or after postoperative day 5 (POD 5). Intraoperative variables, related to postoperative morbidity, were also collected. Blood loss was recorded as binary classification (≥ 400 or less). Besides, surgical approach (laparoscopy or laparotomy), extent of resection (left or right) and operation time were used in this study.

### 2.3. CT image

Preoperative contrast-enhanced CT images with three phases (Non-contrast, arterial phase and venous phase) were used for this model.

CT scans were performed from three manufacturers: Siemens, General Electric (GE) and United Imaging Healthcare (UIH).

The CT scans were acquired using a slice collimation of 5/7 mm, a matrix of 512 × 512 pixels, and an in-plane resolution of 0.516–0.975 mm. Each multi-phase CT image consists of three phases before and after the injection of contrast agent. A Non-Contrast scan is performed before injecting the contrast agent. The post-injection phases include the arterial phase (25–40 s after the injection) and the portal venous phase (60–80 s after the injection). For each patient, all slices containing lesions were used to construct the image dataset.

### 2.4. Outcomes

The primary outcome was PHLF, as defined by the International Study Group of Liver Surgery (ISGLS) as an increased INR (or need of clotting factors to maintain normal INR) and hyperbilirubinemia (according to the normal cut-off levels defined by the local laboratory) on or after postoperative day 5 (POD 5) (7).

### 2.5. Deep learning model development

#### 2.5.1. Data pre-processing

**Figure 1:**
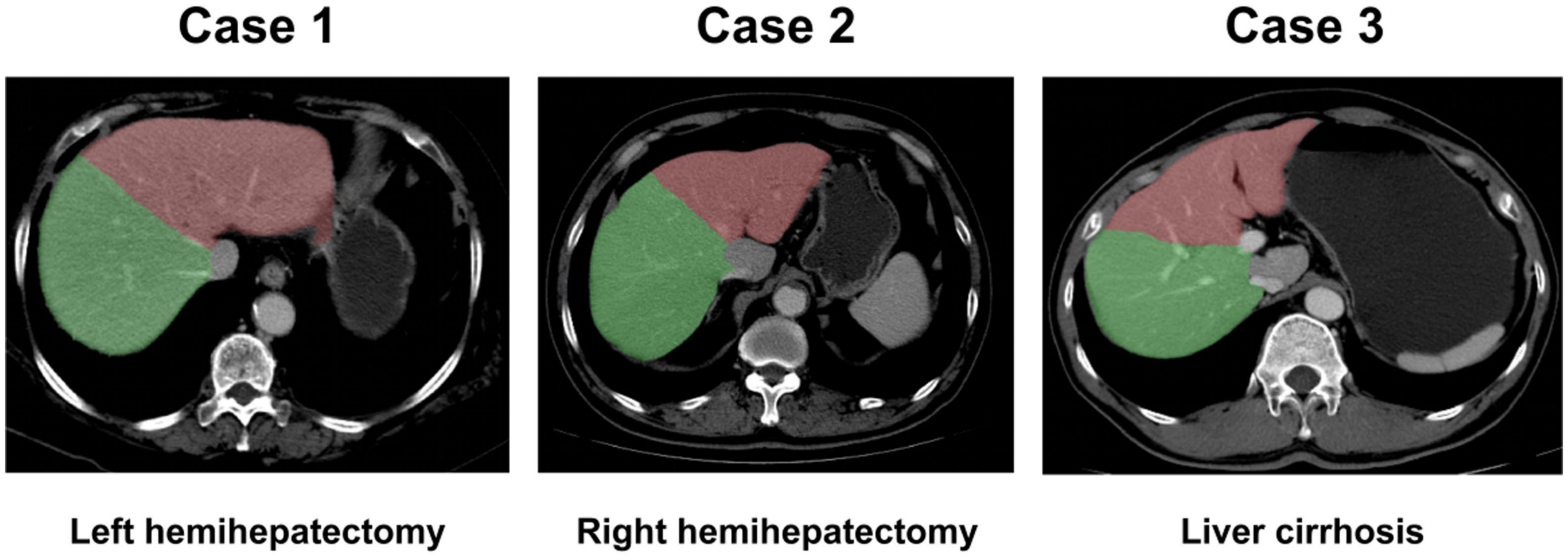
Liver segmentation images. Liver could be segmented automatically. Case 1: left hemihepatectomy; Case 2: right hemihepatectomy; Case 3: liver with cirrhosis. Green region: right liver lobe; Red region: left liver lobe.

In this study, we first extracted liver regions from input liver CT images using via an in-house trained liver segmentation model (Figure 1). This process can be replaced by manual region of interest (ROI) cropping using commercial image annotation tools. Then, the extracted image patches were resized to 16 × 128 × 128 due to different sizes of liver regions in the images. Finally, during training, we randomly cropped 12 × 112 × 112 from the resized image patches and mirrored the crop images to augment the data.

#### 2.5.2. Model architecture framework

**Figure 2:**
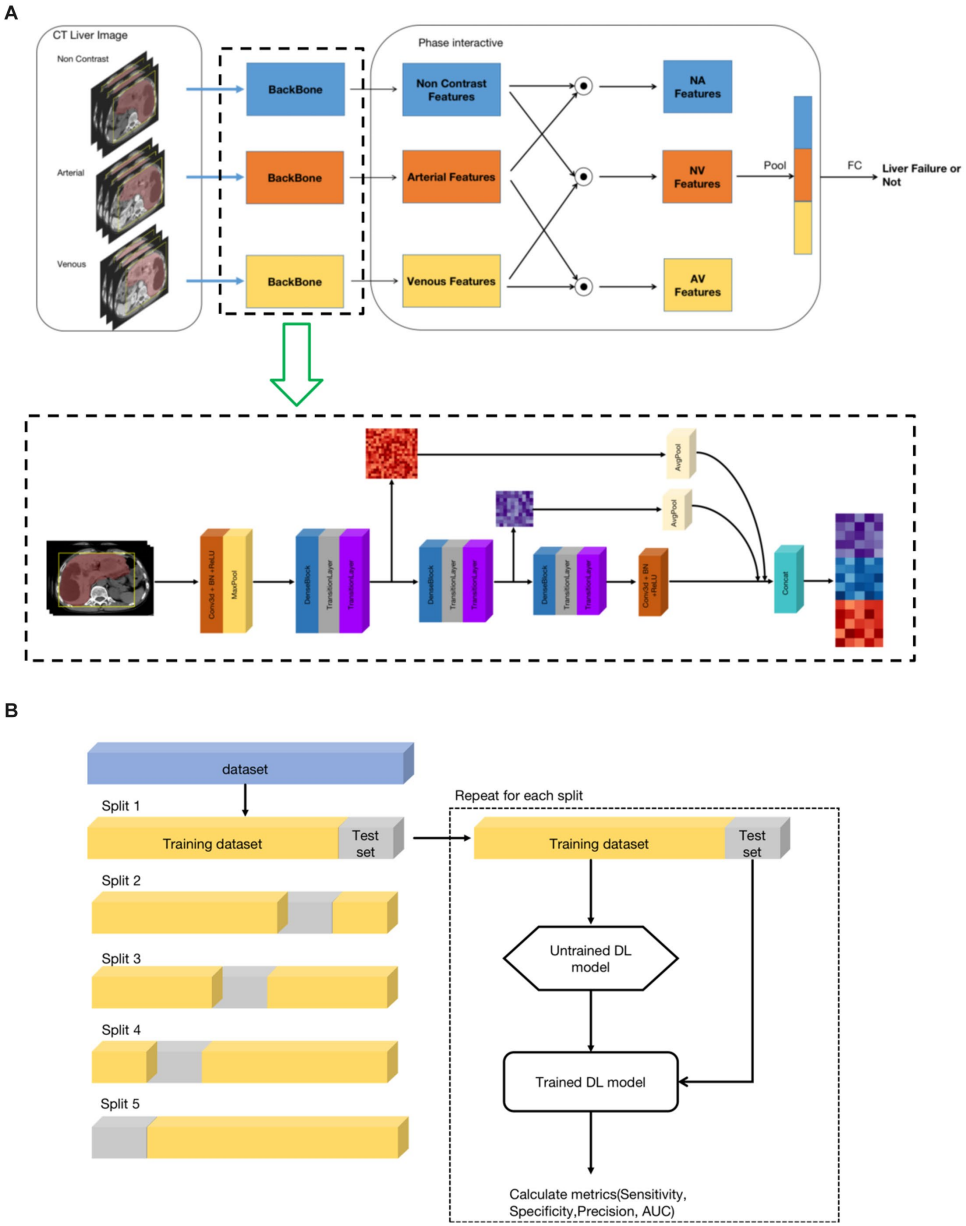
The architecture of the deep learning model. (A) Model architecture framework and implementation details. **(B)** Training and validation of the models. A 5-fold cross-validation technique was used.

For each liver CT examination, there were three phases, including non-contrast phase, arterial phase, and venous phase. To leverage the multi-phase information, we developed a three-phase model framework that utilized the non-contrast, arterial and venous phases of the liver region as inputs. The framework consisted of three stages. The first stage was a feature extraction backbone, which aimed to generate feature maps for the liver region of each of the three-phases. In the second stage, phase-interaction feature fusion was performed by obtaining correlation features between different pairs of phases through the Hadamard product operation. The inputs for this stage were three feature maps with dimensions of 92(C) × 12(D) × 112(H) × 112(W). The resulting correlation feature maps were then passed through 3D average pooling layers, resulting in three one-dimensional feature vector of size 92. In the third stage, the pooled feature vectors were concatenated into a vector of size 276, which was then connected to a fully connected layer to obtain the final classification result, indicating whether the patient had liver failure or not. The overall pipeline of the proposed model framework is illustrated in Figure 2A. It is worth mentioning that precise registration of the three-phase 3D images of the liver region is not necessary.

#### 2.5.3. Model implementation details

As shown in Figure 2A, the feature extraction backbone of the first stage of the framework was based on 3D convolution layers. It was composed of three similar structures, consisting of one dense neural network block, one transition layer, and one SE layer. The dense neural network block origins from the DenseNet (26), inspired by the ResNet (27), which bypassed information from one layer to the next layer via identity connections. The DenseNet architecture distilled shortcut insights into a simple connectivity pattern: ensuring maximum information flow between layers within layers. In this mode, each layer obtained additional inputs from preceding layers and passed its own output to subsequent layers. The DenseNet improved information flow, alleviated the gradient-vanishing problem, enhanced feature reuse, and substantially reduced the number of parameters. The transition layers consisted of 1*1*1 3D convolution and max-pool layer, which reduced the feature map, including reducing the number of feature channels and the size of the feature map. The SE layer used the Squeeze-and-Excitation (SE) block (28), which explicitly modeled the inter-dependencies between channels. Using SE blocks, the network learned to selectively strengthen discriminative features and suppress less informative ones. Finally, the feature map outputs of the three sub-structures of the first stage were concatenated by average pooling to combine high-level and low-level features, thereby improving the feature representation.

#### 2.5.4. Training and validation of the models

In this study, to evaluate the proposed method, we used a 5-fold cross-validation technique. In detail, the dataset was split into 5 folds of equal size, and each fold was used as a test set once, while the other folds were temporarily combined to form a training set. Performance metrics on the test set were then calculated and stored. The process was repeated for the number of folds that have been generated. In each iteration, a new model was trained and tested. At the end of the 5-fold cross-validation run, the collected metrics of the 5 generated DL models were summarized. Finally, the following metrics were calculated: sensitivity, specificity, precision and area under the curve receiver operator characteristic (AUC-ROC). The illustration of the 5-fold cross validation is show in Figure 2B.

### 2.6. Statistical analysis

AUC are created by varying the threshold of the predicted probability and plotting the true positive rate (sensitivity) against the false positive rate (1-specificity). Accuracy, precision, sensitivity, and specificity are also used to evaluate the performance of DL model. All statistical tests use two-tailed tests and *p*-values less than or equal to 0.05 will be considered statistically significant. Statistical analysis was conducted using Python version 3.7.6.

## 3. Results

### 3.1. Characteristics of patients

265 patients with liver CT examination who underwent hemihepatectomy from SRRSH were analyzed, including 170 patients with left liver and 95 patients with right liver. Mean age was 60 ± 13 years. Liver function was evaluated, with only 14 patients (5.3%) classified as Child-Pugh B. 87 patients had viral hepatitis, and 60 patients had liver cirrhosis. The diagnosis was divided into 6 types: 92 (34.7%) hepatocellular carcinoma, 58 (21.9%) intrahepatic cholangiocarcinoma, 5 (1.9%) liver metastases, 27 (10.2%) benign tumor, 78 (29.4%) hepatolithiasis and 5 (1.9%) other liver diseases. 78 (29.4%) patients received open liver resection, and 187 (70.6%) patients with laparoscopic liver resection. 83 (31.3%) patients had intraoperative blood loss more than 400 mL (Table 1). More data was showed in Supplementary Table S1.

**Table 1:** Baseline characteristics and Posthepatectomy liver failure.

Variables	PHLF grade				*p*
	No (*n* = 174)	Yes (*n* = 91)			
		A (*n* = 38)	B (*n* = 45)	C (*n* = 8)	
Age (years)	59.2 ± 12.6	59.6 ± 12.8	59.7 ± 12.6	58.4 ± 13.3	0.076
Gender, male/female	87/87	25/13	31/14	5/3	0.008
Viral hepatitis	40	20	22	5	<0.001
Cirrhosis	24	14	20	6	<0.001
Child-Pugh (A/B/C)	166/8/0	37/1/0	41/4/0	7/1/0	0.566
Diagnosis					<0.001
HCC	42	20	24	6	
ICC	32	11	13	2	
liver metastases	3	0	2	0	
Benign tumor	25	1	1	0	
Hepatolithiasis	67	6	5	0	
Others	5	0	0	0	
ASA (1/2/3)	7/161/6	0/38/0	1/42/2	1/6/1	0.923
Surgical approach					0.105
Laparoscopy	129	32	22	4	
Laparotomy	45	6	23	4	
Extent of resection					<0.001
Left hemihepatectomy	137	17	15	1	
Right hemihepatectomy	37	21	30	7	
Operation time (min)	244 (85–480)	246 (120–420)	281 (75–485)	281 (140–430)	<0.001
Blood loss (ml)					<0.001
≥400	41	10	28	4	
<400	133	28	17	4	

PHLF, posthepatectomy liver failure; HCC, hepatocellular carcinoma; ICC, intrahepatic cholangiocarcinoma; ASA, The American Society of Anesthesiologists. *p* value, PHLF group vs no PHLF group.

### 3.2. Posthepatectomy liver failure

Of all the patients, 91 (34.3%) patients developed PHLF (grade A: *n* = 38; grade B: *n* = 45; grade C: *n* = 8, Table 1). There was no significant difference between PHLF group and no PHLF group on age, ASA, Child-Pugh score and surgical approach. Sex, Viral hepatitis, liver cirrhosis, diagnosis, operation time, blood loss, and extent of resection were associated with PHLF (Table 1, *p* < 0 0.050 for all).

### 3.3. Performance of the DL model on prediction of PHLF

**Figure 3:**
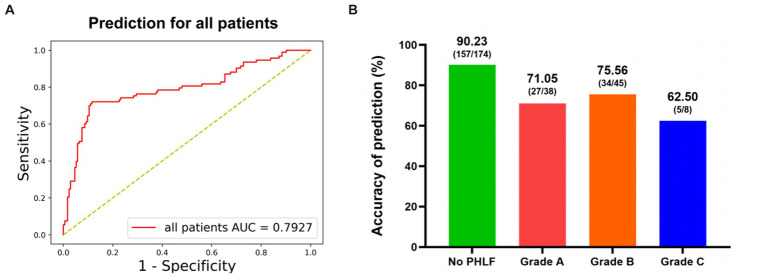
The performance of DL model for prediction of PHLF. **(A)** Receiver-operating characteristic curves and AUC of the predictive models of PHLF for all patients. **(B)** The accuracy for different PHLF grades.

The performance of the DL model on prediction of PHLF was good, with an accuracy value of 84.15% and an AUC value of 79.27% (Figure 3A). The sensitivity was 72.53% (66/91). The specificity was 90.23% (157/174). The precision was 79.52% (66/83) (Table 2).

**Table 2:** Prediction performance for PHLF.

Datasets	Sensitivity (%)	Specificity (%)	Precision (%)	Accuracy (%)
All cases	71.43 (66/91)	89.66 (157/174)	79.52 (66/83)	84.15 (223/265)
Left hemihepatectomy	78.79 (26/33)	91.97 (126/137)	70.27 (26/37)	89.41 (152/170)
Right hemihepatectomy	67.24 (40/58)	81.08 (31/37)	86.96 (40/46)	74.74 (71/95)
Liver with mass	68.75 (55/80)	84.31 (86/102)	77.46 (55/71)	77.47 (141/182)
With liver cirrhosis	77.50 (31/40)	80.00 (16/20)	88.57 (31/35)	78.33 (47/60)
With viral hepatitis	76.60 (36/47)	85.00 (34/40)	85.71 (36/42)	80.46 (70/87)

PHLF, posthepatectomy liver failure; AUC, the area under receiver operating characteristic curve.

The prediction performance of PHLF in different grades was showed in Figure 3B. The accuracy was 71.05% in PHLF Grade A. In patients with severe PHLF (Grade B and Grade C), the accuracy was 73.58% (Grade B: 75.56%, Grade C: 62.50%).

### 3.4. Subgroup analysis

#### 3.4.1. Performance for different extent of resection (left or right hemihepatectomy)

**Figure 4:**
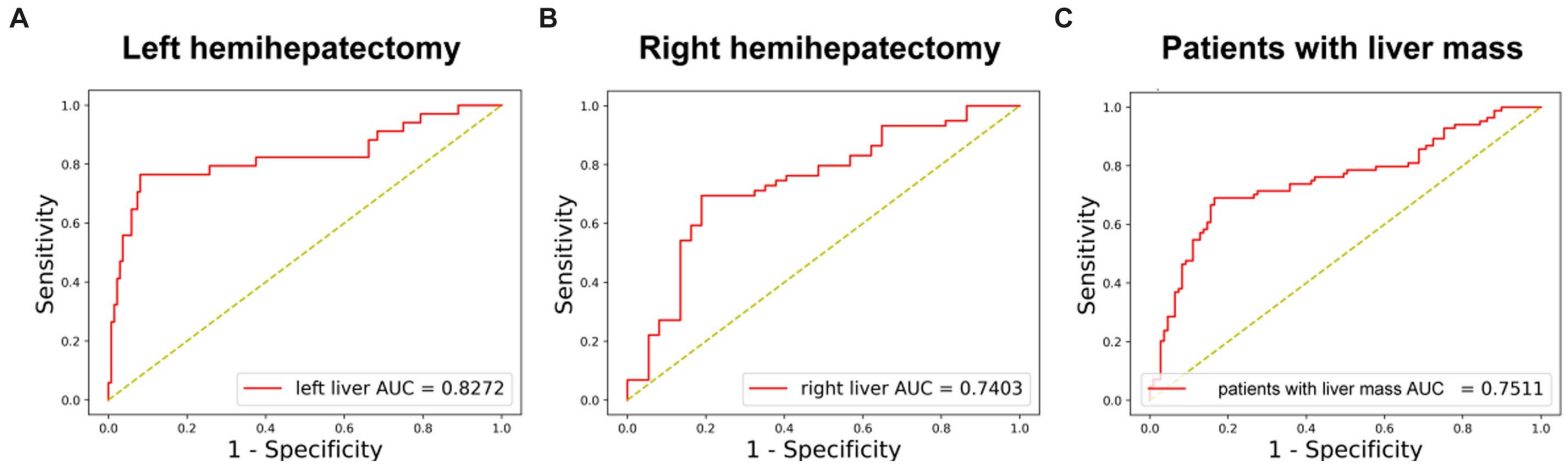
The performance of DL model in subgroups. **(A)** Receiver-operating characteristic curves and AUC of the predictive models of PHLF for left hemihepatectomy patients. **(B)** Receiver-operating characteristic curves and AUC of the predictive models of PHLF for right hemihepatectomy patients. **(C)** Receiver-operating characteristic curves and AUC of the predictive models of PHLF for patients with liver mass (HCC, ICC, liver metastasis or benign tumor).

In 170 left hemihepatectomy cases, the performance of the DL model was better than that in right hemihepatectomy cases (left vs. right: accuracy 89.41% (152/170)vs. 74.74% (71/95); AUC 82.72% vs. 74.03%) (Figures 4A,B). The sensitivity was 78.79% (26/33) inleft hemihepatectomy cases, and 68.97% (40/58) in right hemihepatectomy cases. The specificity was 91.97% (126/137) in the left, 83.78% (31/37) in the right. The precision was 70.27% (26/37) in the left, 86.96% (40/46) in the right.

#### 3.4.2. Performance for patients with liver mass

In 182 patients with liver mass (hepatocellular carcinoma, intrahepatic cholangiocarcinoma, liver metastases and benign tumor), the accuracy was 77.47% (141/182), and AUC was 75.11% (Figure 4C).

#### 3.4.3. Performance for patients with liver cirrhosis or viral hepatitis

The accuracy was 78.33% (47/60) in patients with liver cirrhosis and 80.46% (70/87) with viral hepatitis.

All the results were showed in Table 2.

## 4. Discussion

PHLF is responsible for more than 60% of mortalities after hepatectomy. There were several researches for development of PHLF predictors. Previous study reported that PLT count was related to postoperative liver regeneration, postoperative liver function recovery, and PHLF risk (29, 30). The“50–50 criteria,” an predictor of PHLF and mortality, predicts>50% mortality rate if prothrombin time < 50% and serum bilirubin ≥50 μmoL/L on POD 5 (4). Indocyanine green retention test at 15 min (ICG-R15) is another tool used to evaluate liver quality (12). Mathieu Prodeau et al. (6), reported an ordinal PHLF prediction model for patients with cirrhosis based on 3 variables (i.e., platelet count, RTLV and ITT laparoscopy). Yangling Peng et al. (25), showed a nomogram based on CT–derived extracellular volume for the prediction of PHLF in patients with HCC. Rong-yun Mai et al. (24), developed and validated a multivariate deep learning model based on baseline characteristics, laboratory indicators and surgical situation for predicting the risk of severe PHLF in patients with HCC who underwent hemihepatectomy.

In this study, we first investigated whether PHLF could be predicted by deep learning model based on preoperative enhanced CT. We successfully established a deep learning model and found that it was useful to predict PHLF after hemihepatectomy (precision: 83.40%, AUC: 79.26%). Thus, our findings and the deep learning model may help to select patients who need hemihepatectomies and do a better preparation before operation. In addition, some preoperative characteristics including viral hepatitis, liver cirrhosis, diagnosis and extent of hemihepatectomy were important factors with PHLF. In patients with liver masses, the model showed a performance with AUC 75.10% and precision 77.47% (141/182), sensitivity 68.75% (55/80), specificity 84.31% (86/102). In China, there are many liver resection cases with chronic hepatitis or liver cirrhosis. So, we also evaluated the performance of this model in patients with hepatitis or liver cirrhosis and had a good performance with the precision (chronic hepatitis: 80.46% (70/87); liver cirrhosis: 78.33% (47/60)). In different extent of resection, the result showed that the predictive ability in left hemihepatectomy was better than that in right hemihepatectomy (precision: 89.41% vs. 72.63%; AUC: 82.72% vs. 74.03%). These results suggested that deep learning model could be useful for prediction of PHLF based on CT images, and multiple characteristics were related to the prediction performance of the model.

This model showed a good performance for prediction of PHLF after hemihepatectomy (precision: 83.40%), and it could help to improve the selection of patients with the best risk–benefit profiles for hemihepatectomy. The patients with high PHLF risk, which were selected by the model, could receive other options such as portal vein embolization (PVE), associating liver partition and portal vein ligation for staged hepatectomy (ALPPS), radiofrequency ablation (RFA) or transcatheter arterial chemoembolization (TACE) (31–33). Besides, the model could help surgeons modify the perioperative treatment plan for the high PHLF risk patients. Enhanced recovery after surgery (ERAS) and prehabilitation could be used for high risk patients who were selected by the model. Furthermore, the prediction data of the model could also help patients understand the risk–benefit before surgery, and be useful for preoperative conversation and seeking informed consent.

There are some limitations in our study. First, the more cases from different centers were needed. Second, the precision and AUC in right hemihepatectomy were not as good as that in left hemihepatectomy. Possible reasons included small number of right hemihepatectomy cases and more complicated surgical situation. Third, this study was not designed to predict other outcomes such as 30-day, 90-day or 1-year mortality. Forth, patients’ basic characteristics, laboratory indicators and surgical situation were not included in our model. Thus, multimodal algorithm based on more effective medical data, would be developed to achieve adequate performance. Fifth, a prospective and multicenter study is required to clarify the reliability and adaptability of the deep learning model.

In conclusion, this preliminary study obtains a deep learning model for prediction of PHLF (as defined by the International Study Group of Liver Surgery (ISGLS)), which can be accomplished with a high precision based on preoperative contrast-enhanced CT images. However, further study would be necessary to improve performance for prediction of PHLF.

## Data availability statement

The original contributions presented in the study are included in the article/Supplementary material, further inquiries can be directed to the corresponding authors.

## Ethics statement

Ethical review and approval was not required for the study on human participants in accordance with the local legislation and institutional requirements. Written informed consent from the participants was not required to participate in this study in accordance with the national legislation and the institutional requirements.

## Author contributions

HY, YY, XL, and XX contributed to the study concept and design. HY, YR, XX, and YT contributed to acquisition of data. ZiX, SW, and XLiu contributed to analysis and interpretation of data. XX, HY, and XLiu contributed to writing, reviewing, and approval of the final version of this work. All authors contributed to the article and approved the submitted version.

## Funding

This research was supported in part by National Key Research and Development Program of China (No. 2019YFC0118100), the Zhejiang Provincial Key Research & Development Program (No. 2020C03073), Medical Health Science and Technology Project of Zhejiang Provincial Health Commission (2020RC069).

## Conflict of interest

The authors declare that the research was conducted in the absence of any commercial or financial relationships that could be construed as a potential conflict of interest.

## Publisher’s note

All claims expressed in this article are solely those of the authors and do not necessarily represent those of their affiliated organizations, or those of the publisher, the editors and the reviewers. Any product that may be evaluated in this article, or claim that may be made by its manufacturer, is not guaranteed or endorsed by the publisher.
